# Le coût médical direct de la prise en charge hospitalière des fractures de l’extrémité supérieure du fémur

**DOI:** 10.11604/pamj.2017.27.133.6731

**Published:** 2017-06-20

**Authors:** Abdelghani El Ayoubi, Kevin Parfait Bienvenu Bouhelo, Hachem Chafik, Mohammed Nasri, Mohammed El idrissi, Mohammed Shimi, Abdelhalim El ibrahimi, Abdelmajid Elmrini

**Affiliations:** 1 Service de Chirurgie Ostéoarticulaire B4, CHU Hassan II, Université Sidi Mohammed Ben Abdellah, 3000 Fès, Maroc

**Keywords:** Santé publique, épidémiologie, fracture, l´âge, le coöt, prise en charge, Public health, epidemiology, fracture, age, costs, management

## Abstract

Les fractures de l'extrémité supérieure du fémur sont des fractures graves du fait de leur morbidité et de leurs conséquences sociales et/ou économiques. Elles font l'objet de plusieurs études à l'échelle mondiale concernant leurs prises en charge thérapeutique, leurs évolutions et leurs préventions. En raison du vieillissement de la population et de l'allongement de l'espérance de vie, l'augmentation de l'incidence de cette pathologie paraît inéluctable et posera à terme un véritable problème de santé publique dont l'importance sera encore majorée par la nécessité d'une maîtrise des dépenses de santé. Les résultats de ce travail montrent que l'âge moyen de survenue d'une fracture de l'extrémité proximale du fémur est de 68,13 ± 16,9 ans, et que la prédominance a été masculine avec un sex-ratio de 1,14. Les fractures per trochantériennes ont représenté 69,4% des cas. Le coût médical direct hospitalier des fractures de l'extrémité supérieure du fémur au CHU Hassan II est de 387 714,38 £ chez 222 cas, avec un coût moyen de 1757,4 £, dont les frais liés au séjour ont représenté la majorité des dépenses avec 77% du coût total. Pour diminuer le coût de la prise en charge de cette pathologie, il est souhaitable de sensibiliser le personnel sur le coût des consommables à fin d'adapter le comportement le plus économique. Et aussi il faudrait limiter au maximum la durée de séjour car elle seule permet de réduire les dépenses liées au frais du personnel et l'hôtellerie.

## Abstract (en)

Fractures of the upper extremity of the femur are serious because of their morbidity and social and/or economic consequences. They have been the subject of several studies of world literature concerning their hospital treatment, evolution and prevention. The increase in the incidence of this pathology seems unavoidable due to population ageing and to the lengthening life expectancy; it is posing a real long-term public health problem whose importance will be further increased by the need to control health care costs. The results of this study show that the average age of onset of fracture of the proximal extremity of the femur is 68,13 ± 16.9 years, with a male predominance and a sex ratio of 1.14. In our study pertrochanterian fractures represented 69.4% of cases. Direct medical costs of the hospital treatment of fractures of the upper extremity of the femur at the Hassan II University Hospital were £387 714,38 in 222 cases, with an average cost of £1757,4 , including costs for patient’s stay in hospital, which represented the majority of expenses ( 77% of total costs). It is desirable to raise staff awareness of the costs of consumables in order to reduce treatment costs and to adopt cost-oriented behaviour. Length of stay should be limited to the maximum extent because it only allows to reduce staff and accommodation costs.

## Introduction

Les fractures de l'extrémité supérieure du fémur (FESF) sont graves du fait de leur morbidité et de leurs conséquences sociales et économiques. Elles sont rencontrées généralement chez les sujets âgés. Elles font l'objet de nombreuses études à grande échelle concernant leur prise en charge thérapeutique et leur évolution à court, moyen et long terme. Leur épidémiologie est d'un abord simple, peu onéreux, mais surtout leurs conséquences sont particulièrement sévères : impotence, dépendance, mortalité. En raison du vieillissement de la population et de l´allongement de l´espérance de vie, l´augmentation de l´incidence de cette pathologie paraît inévitable et posera à terme un véritable problème de santé publique et constitue donc un enjeu économique de premier ordre. Le coût individuel de prise en charge de cette pathologie croit avec le développement médicotechnique avec l'avènement de nouvelles thérapeutiques, sa prévention passe par le contrôle des facteurs de risques et par l'évaluation médicale et médico-économique des moyens disponibles. La détermination des coûts par pathologie permettra d´établir le budget de fonctionnement des services hospitaliers selon les dépenses liées au traitement de ces pathologies.

## Méthodes

Il s'agissait d'une étude rétrospective étalée sur deux ans du 1^er^ janvier 2011 jusqu'au 31 Décembre 2012, colligé au service de chirurgie traumato-orthopédique B4 au CHU HASSAN II de Fès-MAROC, elle a inclus 222 patients admis tous pour prise en charge des fractures de l'extrémité proximale du fémur. Le coût médical de prise en charge hospitalière des patients était défini comme étant la somme des coûts de séjours, des bilans radiologiques, des bilans biologiques, des fongibles, et de l'acte opératoire Tableau 1. Le coût du matériel d'ostéosynthèse n'a pas été pris en considération lors de notre étude puisqu'il était fourni par des sociétés extrahospitalières. Le recueil des données a été réalisé en se basant sur les données des dossiers médicaux des patients, et les factures fournis par le BAF (Bureau des Admissions et Facturation du Centre Hospitalier Universitaire Hassan II). Tout les coûts on été calculés au prix du 2012 au terme de devise national (DHM) et ont été convertis en EURO selon un taux de change fixe (1DHM= 0,092330 EURO) et leur analyse a été effectuée à l'aide du logiciel Epi Info 7.

**Tableau 1:** Coût des examens préopératoires des patients

l'examen	coût(DH)	Coût(£)
Radiographie de la hanche face	112,5	10,387125
radiographie du thorax face	120	11,0796
numération formule sanguine(NFS)	72	6,64776
ionogramme complet (urée, créatinine,Na+; K+;Cl-…)	144	13,29552
TP	36	3,32388
TCK	36	3,32388
groupage sanguin	45	4,15485
glycémie	25	2,30825
ECG	60	5,5398
échographie transthoracique (ETT)	500	46,165

## Résultats

**Figure 1:**
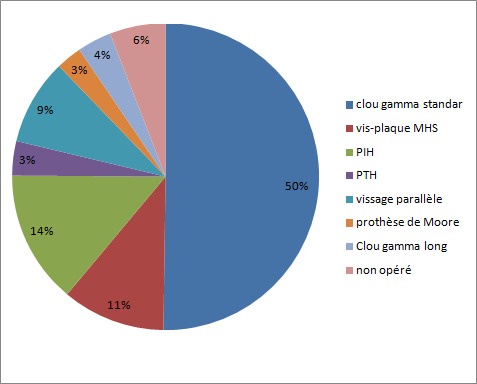
Type de matériel d´ostéosynthèse

L'âge moyen des patients inclus dans notre étude était de 68,13 ±16,9 ans (extrêmes 19 et 98 ans) ; avec une légère prédominance masculine (sex ratio : 1,14). La durée du séjour hospitalier était de 8,9 ± 4,93 jours en moyenne, avec des extrêmes de 1 jour et 35 jours. Dans notre étude 22 patients ont séjournés au service de réanimation pendant une durée variante entre 1 jour et 13 jours, avec une moyenne de 2jr ± 2,65. Au total 5 patients ont décidé lors de leurs séjours. Les fractures pertrochantériennes ont représenté 69,4 % avec 154 cas, et 68 cas présentaient une fracture du col fémoral (30,6%). Les moyens d'ostéosynthèses représentées essentiellement par des tuteurs internes type clou gamma dans le traitement des fractures pertrochantériennes, utilisés chez 111 cas. L'arthroplastie par prothèse intermédiaire de la hanche (PIH) a été utilisée chez 14% des cas et 12 cas (6 %) non opéré en raison des contre-indications à l'anesthésie (Figure 1). Le coût médical direct moyen de la prise en charge hospitalière des patients a été estimé à 1757,4 £, dont les frais liés au séjour hospitalier ont représentaient plus de 77% (Tableau 2).

**Tableau 2:** Coût moyen et total de prise en charge hospitalier des 222 cas étudiés

	**coût moyen (£)**	**coût total(£)**
Coût des examens biologiques	33,73	7488,88
Coût des examens radiologiques	27,15	6028,68
Coût des médicaments	50,02	11054,51
coût des fongibles	98,47	21862
Coût de séjour	1349,7	299633,93
Coût d'acte opératoire	198,31	41646,37
Total	1757,4	387714,38

## Discussion

Le coût médical direct moyen de prise en charge d'une fracture de l'extrémité supérieure du fémur a été estimé selon notre étude à 1757,4 Euro, qu'on peut considérer sous-estimé puisque le coût du matériel d'ostéosynthèse n'a pas été inclus dans nos calculs. L'étude des différents paramètres du coût médical de prise en charge des fractures de l'extrémité proximale du fémur a trouvé que le coût du séjour hospitalier a représenté la majorité du coût total avec plus de 77%, alors que le coût de l'acte opératoire n'a représenté que 10,7 %. Une réduction de la durée du séjour hospitalier des patients va permettre de baisser d'une façon considérable le coût de prise en charge de ces patients, ce qui est devenu faisable avec la disponibilité du matériel d'ostéosynthèse au sein de la pharmacie du CHU. Les 222 cas inclus dans notre étude ont occupé des lits hospitaliers durant 1285 jours avant l'intervention chirurgicale, avec une dépendance aux soins infirmiers et prolongation de la durée du séjour hospitalier. La disponibilité du matériel et les ressources adéquats et la mise en place d'un programme sanitaire permettront le contrôle du séjour hospitalier de ces patients et de réduire les dépenses liées à leurs prises en charge [1]. La prévention des fractures du fémur proximal par le contrôle des facteurs de risques, représentés essentiellement par l'ostéoporose et les chutes [2–4], va permettre sans doute une réduction du coût médical direct de prise en charge hospitalière de cette pathologie et par conséquence diminuer leurs charges financières. De nombreux types de molécules ont démontré leur capacité à accroitre la masse osseuse et ainsi à réduire le risque de fracture. C'est le cas de la calcitonine, des sels de calcium ou de fluor, des diphosphonates, de la vitamine D. Toutefois, c'est incontestablement la supplementation calcique [2] et l'apport vitaminique [5] qui génèrent les économies les plus importantes. Ces mesures préventives seront utiles après une analyse coût-bénéfices qui mettra en valeurs chaque moyen [6, 7]. La mesure effective des coûts est complexe. Le coût médical direct hospitalier est le plus calculer dans la plupart des études: il est représenté par la consommation de soins lors de l'hospitalisation, des médicaments, et l'ensemble d'investigations diagnostic et thérapeutiques (laboratoires, radiologies, fongibles, bloc opératoires). Ceci conduit à apprécier difficilement le coût exact des traumatismes de la hanche. Ainsi on peut avoir une sous ou surestimation du coût de prise en charge des fractures de l'extrémité supérieure du fémur. En effet, le calcul du coût des médicaments se base sur la feuille des prescriptions et la factures fournis par le bureau de facturation et des admissions(BAF) du CHU mais ne tient pas compte des incidents fréquents tels qu'une perte de flacon, et les erreurs liées au système informatisé de la pharmacie central (divergence). Les frais généraux sont difficiles à déterminer de manière exacte vu leur diversification, en plus, on estime aussi les frais du personnel qui ne sont pas toujours mesurables car ils ne sont pas toujours monétaires.

## Conclusion

Les fractures de l'extrémité supérieure du fémur ont des conséquences financières majeures sur les budgets hospitaliers non seulement en matière des frais monétaires mais aussi sur les coûts indirects. Dans notre étude, nous nous sommes basés que sur les frais impliqués directement dans la prise en charge des patients victime d'une fracture de l'extrémité supérieure du fémur, pendant leurs séjours intra-hospitaliers. L'adaptation d'un comportement économique en tenant compte du coût des consommables permettra de diminuer le coût de prise en charge de cette pathologie, et aussi il faudrait limiter au maximum la durée de séjour car elle seule permet de réduire les dépenses liées au frais du personnel et l'hôtellerie.

### Etat des connaissances actuelles sur le sujet

- Les fractures pertrochantériennes sont parmi les fractures les plus fréquentes en traumatologie des sujets âgés ;
- Problème majeur de santé public ;
- Le traitement chirurgical des fractures pertrochantériennes permet un regain de l'autonomie antérieure des patients précocement.

### Contribution de notre étude à la connaissance

- Le coût médical de la prise en charge de cette pathologie augmente avec les progrès que reconnait le domaine médicotechnique ces dernières décennies;
- Le séjour hospitalier représente la partie la plus importante du coût total de la prise en charge de cette pathologie traumatique;
- La disponibilité du matériel d'ostéosynthèse et la sensibilisation du personnel du bon usage des fongibles permettra de réduire considérablement le coût de la prise en charge de ces fractures.

## Conflits d’intérêts

Les auteurs ne déclarent aucun conflit d'intérêts
